# Probabilistic prediction of increased intracranial pressure in patients with severe traumatic brain injury

**DOI:** 10.1038/s41598-022-13732-x

**Published:** 2022-06-10

**Authors:** Priyantha Wijayatunga, Lars-Owe D. Koskinen, Nina Sundström

**Affiliations:** 1 Department of Statistics, Umeå University, Umeå, Sweden; 2 Department of Clinical Science – Neurosciences, Umeå University, Umeå, Sweden; 3 Department of Radiation Sciences, Radiation Physics, Biomedical Engineering, Umeå University, Umeå, Sweden

**Keywords:** Computational neuroscience, Learning algorithms, Neurosurgery

## Abstract

Traumatic brain injury (TBI) causes alteration in brain functions. Generally, at intensive care units (ICU), intracranial pressure (ICP) is monitored and treated to avoid increases in ICP with associated poor clinical outcome. The aim was to develop a model which could predict future ICP levels of individual patients in the ICU, to warn treating clinicians before secondary injuries occur. A simple and explainable, probabilistic Markov model was developed for the prediction task ICP ≥ 20 mmHg. Predictions were made for 10-min intervals during 60 min, based on preceding hour of ICP. A prediction enhancement method was developed to compensate for data imbalance. The model was evaluated on 29 patients with severe TBI. With random data selection from all patients (80/20% training/testing) the specificity of the model was high (0.94–0.95) and the sensitivity good to high (0.73–0.87). Performance was similar (0.90–0.95 and 0.73–0.89 respectively) when the leave-one-out cross-validation was applied. The new model could predict increased levels of ICP in a reliable manner and the enhancement method further improved the predictions. Further advantages are the straightforward expandability of the model, enabling inclusion of other time series data and/or static parameters. Next step is evaluation on more patients and inclusion of parameters other than ICP.

## Introduction

Traumatic brain injury (TBI) is a type of head injury that causes temporary or permanent alteration in brain functions. It is one of the major health problems currently worldwide. Some of the main causes of TBI are automobile and sports accidents and falls^1^. The correct identification of clinical state of patients suffering severe TBI is often crucial for subsequent medical treatments and for reduction of secondary injuries following the initial insult. One such secondary injury is elevated intracranial pressure (ICP) which may result in decreased microcirculation, cerebral ischemia and infarcts^2^. Monitoring of ICP is recommended in patient with severe TBI with a Glasgow Coma Scale score of 3–8 from the time of arrival to the intensive care unit (ICU) until the clinical state improves, or in worst case, until death occurs^3,4^. An approximate mean treatment time in the ICU is two weeks. Consensus guidelines from the Brain Traumatic Foundation state that a critical threshold for which treatment action should be initiated to maintain ICP below is 22 mmHg^5^, since levels above this are associated with poor clinical outcomes. However, a limit of 20 mmHg is also common in clinical practice, including at our own hospital, and this limit was considered in the current study^6,7^.

During treatment in the ICU, it is difficult to know in which patients the clinical state will remain at a stable level and in which one’s ICP will increase rapidly to dangerous levels. Thus, the possibility to predict future increases in ICP, and treat them to avoid additional pressure load on the brain, would be clinically very valuable. Further, it is of outmost importance for the clinician to act before a secondary negative event is established.

Apart from ICP, the setup for multimodal monitoring in the ICU generally includes an extensive battery of other high frequency physiological parameters such as mean arterial blood pressure (ABP), cerebral perfusion pressure (CPP), echocardiography, oxygenation, temperature, and respiration. However, this kind of extended monitoring is not feasible in every part of the world, mainly due to lack of resources. Also, although the information generated from multimodal monitoring equipment aids in the treatment process in the ICU, it is generally too complex and diverse to be fully understood in real time by the treating clinician^8^. Thus, computational aids based on simple and relatively easily obtainable data would be advantageous, but the possibility of incorporating richer data streams as well opens for even better prediction models and further understanding of the physiological processes involved during treatment in the ICU.

During the last decades, computational power and methods based on artificial intelligence and machine learning have increased tremendously. It is anticipated that these statistical predictive methods will have a profound impact on health care in general, and on the treatment in the ICU in particular, since the management is data-intense and often based on multimodal monitoring^9,10^. In a recent review, it was shown that out of the 258 papers applying some kind of machine learning method to data routinely collected in the ICU, the most common applications were predicting complications (29.8%), predicting mortality (27.1%), improving prognostic models (16.7%) and classifying sub-populations (11.2%)^11^. Thus, there is a need for models that can predict adverse events in real-time during patient monitoring.

A model for ICP prediction in patients with TBI treated in the ICU has previously been presented^9^. This model predicted the occurrence of episodes (i.e., at least 10 consecutive minutes) with ICP ≥ 30 mmHg with good accuracy. According to the recommendations from the Brain Traumatic Foundation and the clinical guidelines at Umeå University Hospital, it would be preferable to have a system set out to warn already at ICP > 20 mmHg. Also, the general “dose” regarding increased ICP has been shown to correlate with clinical outcome^12–14^, and mean ICP over 10-min intervals, as opposed to continuous values during the same period, has the potential to reflect this burden while still being resilient to transient phenomena such as coughing. A computational framework has also been proposed to predict prolonged intracranial hypertension^15^, and currently popular machine learning methods have been applied to ICP prediction^16^.

The aim of this study was to develop a probabilistic tool with explainable outputs to predict future ICP levels of individual TBI patients in the ICU within the next hour to come. The model should be based on ICP alone, to be applicable in most ICU facilities, but expandable to incorporate other time series and static patient information as well.

## Methods

### Patients

The probabilistic model presented and evaluated in this paper is based on ICP time series data collected from 29 patients suffering from severe TBI and treated with neuro-intensive care at Umeå University Hospital. All patients were prospectively recruited from January 2015 to December 2017. Inclusion criteria were persons of all ages with a clinical diagnosis of severe TBI and an indication for CT that presented to the hospital within 24 h of injury. Informed consent was obtained according to local and national requirements. Patients with severe preexisting neurological disorders that would confound outcome assessments were excluded. All patients were treated by an ICP oriented regimen referred to as a modified version of the Lund concept^17^. This includes surgical removal of volume expanding lesions, optimization of microcirculation and metabolism by sedation, intubation and mechanical ventilation keeping a normal PO_2_ and PCO_2_. A normal osmotic and hydrostatic pressure is desirable to counteract brain edema. Ventriculostomy is used as needed. Osmotic agents, such as mannitol, are avoided due to the risk of opening of the blood–brain-barrier and a secondary increase in edema. Action is taken to counteract the sympathetic nervous system storm to dampen the inflammatory response and to optimize the hydrostatic pressure over the cerebral capillaries. This approach intends to keep ICP below 20 mmHg and in some cases allowing CPP down to 50 mmHg, to avoid the negative effects of pressor drugs and hypervolemia. CPP is generally not treated in order to exceed 60 mmHg.

Time series data were collected by the Moberg CNS unit (Moberg Research, Inc., Ambler) as part of the CENTER-TBI study^18^ and an ongoing local study. Sampling frequency was 125 Hz, which was rescaled to minute values. A software developed in Matlab (Matlab 2019b, The MathWorks Inc, Natick, MA, USA) was applied to identify and remove all artifacts lasting longer than three seconds (in total, 2.5% of the data were removed). The artefacts were related to e.g., disconnection of the patient for transport to x-ray and physical management of the patient and/or ICP probe in the ICU. In total, the model building and testing were based on 4018 h of ICP recordings. The limit for dangerously high ICP levels was set to 20 mmHg, i.e., ICP < 20 mmHg was classified as “1” (normal) and ICP ≥ 20 mmHg was classified as “2” (severe). Of the time series data, 81.7% were found to be < 20 mmHg (i.e., “ICP = 1”) and 18.3% were ≥ 20 mmHg (“ICP = 2”). Future ICP levels were predicted for six consecutive 10-min intervals in the next hour from the present time.

### Ethical approval

The study was approved by the Swedish Ethical Review Authority (2014/1473-31/4, 2011-256-31 M, 00-175 and 2013-43-31 M) and followed the World Medical Association Declaration of Helsinki.

### Model description

A probabilistic model for the prediction of future ICP levels of individual patients was developed. The model is generalizable to any number of predictor variables but can be simplified when predictions are to be based on ICP data streams alone. The model is a more general dynamic version of the so-called naïve Bayes model^19^, i.e., both the predictors and the predicted variables can be time series, although some of the predictors can be static random variables as well.

Let ${X}_{i:t}$ denote the observation at time $t$ (discrete, for simplicity) of the *i*th time series, for $t=1, \mathrm{2,3},\dots$ and $i=1,\dots ,p$. Here $p\ge 1$ is the number of time series denoted by $\left\{{X}_{1:t}\right\},\dots ,\left\{{X}_{p:t}\right\}.$ Assume that there are no long-term trends in any of the time series. The model should predict, without loss of generality, all *m* number of time steps into the future from current time $t$ of the 1st time series, i.e., $\left({X}_{1:t+1},\dots ,{X}_{1:t+m}\right)$. For the model to make independent predictions for each of these time steps, it is defined as:

$$P\left({X}_{1:t+k}|{X}_{1:\left[t-{d}_{1},t\right]},\dots ,{X}_{p:\left[t-{d}_{p},t\right]}\right)=\frac{P({X}_{1:t+k}){\prod }_{i=1}^{p}P({X}_{i:\left[t-{d}_{i},t\right]}|{X}_{1:t+k})}{{\sum }_{{X}_{1:t+k}^{^{\prime}}}P({X}_{1:t+k}^{^{\prime}}){\prod }_{i=1}^{p}P({X}_{i:\left[t-{d}_{i},t\right]}|{X}_{1:t+k}^{^{\prime}})}$$

where $k=1,\dots ,m$ and integer ${d}_{i}>0$ for $i=1,\dots ,p.$ Here ${X}_{i:[t-{d}_{i},t]}={(X}_{i:t-{d}_{i}},{X}_{i:t-{d}_{i}+1},\dots ,{X}_{i:t})$ is the history of the *i*th time series ${d}_{i}$ number of time steps back from the present time $t$, for $i=1,\dots ,p.$ The model gives conditional probability of the value of the *k*th time step of the 1st series, given a finite history of each of the time series available.

In this study the simplest version of the above model, where $p=1 \mathrm{ for }{d}_{1}>1$, was evaluated. When mean ICP at a desired time interval into the future, e.g., the *k*th time step from the present time $t,$ is predicted using only the history of ICP itself from the past time $(t-{d}_{1})$ until the present time, it is obtained as

$${x}_{1:t+k}^{*}={ArgMax}_{{x}_{1:t+k}} p({x}_{1:t+k}|{x}_{1:\left[t-{d}_{1},t\right]}).$$

Note that when $k=1,$ the average ICP value for the first 10-min interval from the present time is predicted, when $k=2,$ the second 10-min interval is predicted and so on.

### Prediction enhancement

To achieve a prediction enhancement favoring more accurate predictions of instances of ICP ≥ 20 mmHg (ICP classified as “2”), the *k*th-time step prediction was obtained as

$${x}_{1:t+k}^{*}={ArgMax}_{{x}_{1:t+k}} \left\{\begin{array}{c}\alpha p\left({x}_{1:t+k}=1|{x}_{1:\left[t-{d}_{1},t\right]}\right), \\ p\left({x}_{1:t+k}=2|{x}_{1:\left[t-{d}_{1},t\right]}\right)\end{array}\right\}$$

where $0<\alpha \le 1.$ Here, $\alpha$ acts as a “balancing factor” to compensate for the imbalance of the data. E.g., when $\alpha =0.65$ is selected, if we have $p\left({x}_{1:t+k}=1|{x}_{1:\left[t-{d}_{1},t\right]}\right)=0.6$ and therefore $p\left({x}_{1:t+k}=2|{x}_{1:\left[t-{d}_{1},t\right]}\right)=0.4$, then the prediction is ${x}_{1:t+k}^{*}=2,$ since $0.65*0.6=0.39<0.40.$ The selection of $\alpha$ was based on the equation:

$$T=A{f}_{1}\left(\alpha \right)+w(1-A){f}_{2}(\alpha )$$

where *A* is the fraction of *ICP* = *1*, ${f}_{i}\left(\alpha \right)$ is the gain in prediction accuracy for *ICP* = *i* where *i* = *1,2*, due to $\alpha ,$ and $w\ge 1$ is the weight of *ICP* = *2* compared to that of *ICP* = *1.* For α = 0.50 to 0.85 with increments of 0.05 the value of T (T > 0) was calculated for all six prediction time intervals. The α that maximized T was close to 0.60 for all time intervals. Thus, for obtaining enhanced predictions favoring accuracy in ICP = 2 as opposed to ICP = 1, $w=2$ and $\alpha =0.60$ in this study.

### Training of the model

**Figure 1:**
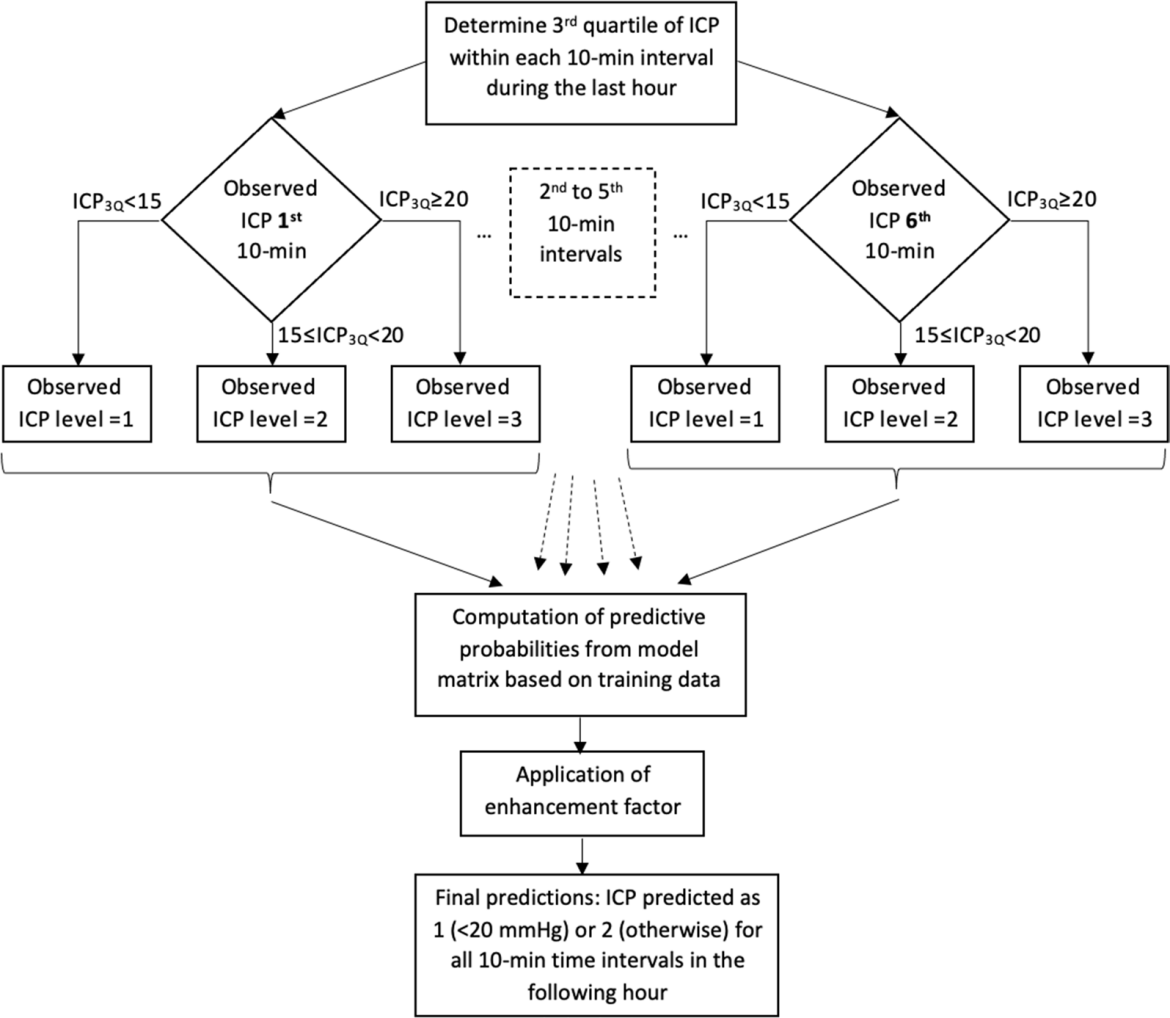
Flow chart for prediction of ICP levels within the next hour to come.

All data were divided into 10-min intervals. Mean ICP of each interval was defined as the response value. The preceding hour of data (i.e., six 10-min intervals) were taken as the predictor values for that specific response value. First, we based our model on the mean and median values of ICP during each 10-min time interval respectively, but once the third quartile was used instead, we found that this improved the prediction accuracy of the model. Therefore, these predictors were discretized into three levels based on their third quartile (ICP_3Q_) such that 1: ICP_3Q_ < 15 mmHg, 2: 15 ≤ ICP_3Q_ < 20 and 3: ICP_3Q_ ≥ 20 mmHg, respectively. Thus, each 10-min interval was categorized as either 1, 2 or 3 depending on the value of the third quartile during that period. Figure 1 shows a flow chart of the prediction scheme.

### Evaluation of the probabilistic model for ICP prediction

Prediction accuracy was evaluated based on a random selection of 10-min intervals from all patients. This was done for all six intervals of the next hour from the present. That is, for each patient, 80% of the 10-min intervals were randomly selected for training the model while the remaining 20% were used as test data. Mean prediction accuracies for each 10-min interval along with their non-parametric bootstrap confidence intervals (CI) were calculated. In a similar manner, the mean prediction accuracies for individual patients were also evaluated. Here, the model was based on continuous ICP time series data from all but one patient, for which the predictions were evaluated.

### Statistical analyses

All model building, training and statistical analyses were performed using the free software R^20^ on an Apple MacBook Pro Retina (Processor: 2.5 GHz Quad-core Intel Core i7, Memory: 16BG, 1600 MHz with macOS Big Sur version 11.3.1).

### Consent to participate

Informed consent was obtained from all patients according to local and national requirements.

## Results

**Figure 2:**
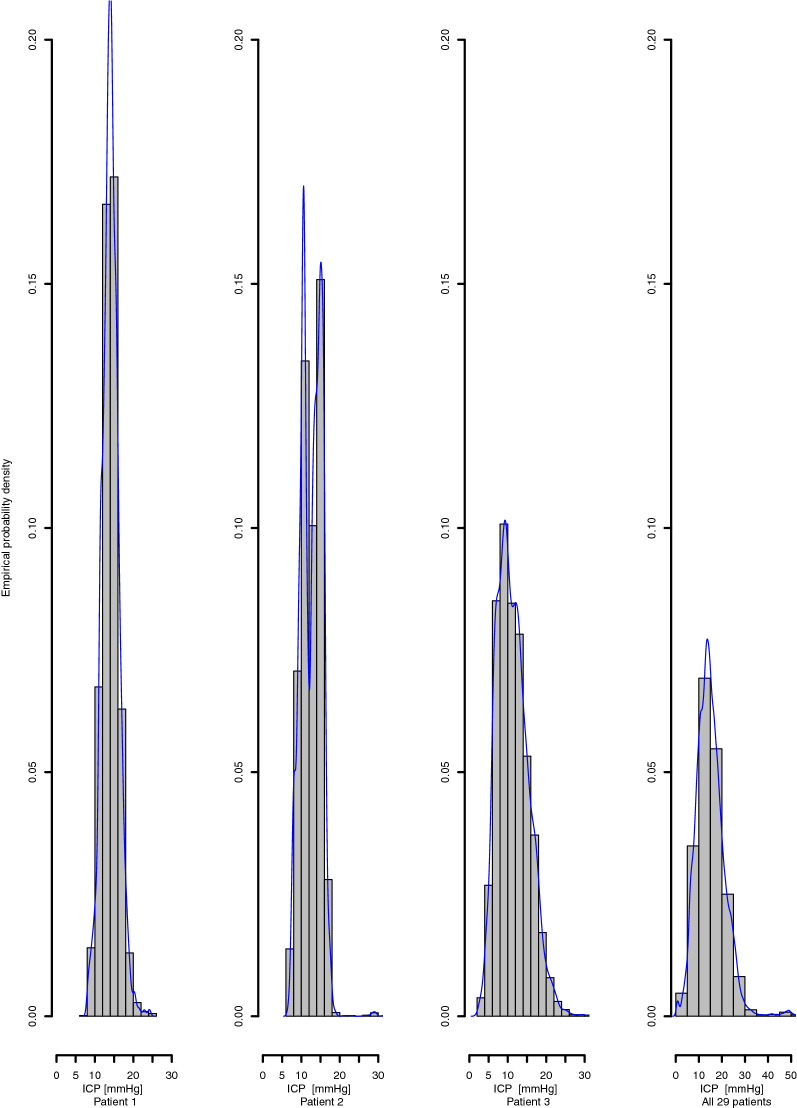
Probability density of ICP. The first three distributions display the probability density of the ICP measurements for three typical patients monitored and treated in the ICU. Glasgow outcome scale extended for these patients were 4, 3 and 6 respectively at 6-months clinical follow up. The rightmost distribution includes the total monitoring time for all 29 patients.

Patient characteristics are described in Table 1. Figure 2 shows the distribution of mean ICP over 10-min intervals for three selected subjects and for all 29 subjects, respectively. The highly imbalanced nature of the data, regarding the prediction threshold of 20 mmHg, is clearly displayed.

**Table 1:** Patient characteristics.

Variable	Value
Median age in years (range)	56 (20–80)
Sex (No. female/male)	7/22
Total monitoring time (h)	4018
Mean (SD) monitoring time per patient (h)	135.7 (29.0)
Mean (SD) ICP for all patients (mmHg)	14.6 (4.2)
6-months mortality [No. (%)]	4 (14)
12-months mortality [No. (%)]	5 (17)

Table 2 shows the mean prediction accuracies and 95% bootstrap CI for test sets of randomly selected instances each 10-min interval in the next hour. Table 3 shows the weighted mean and range for the prediction accuracies of individual patients. The weights were based on the proportion of ICP = 1 and ICP = 2 for each patient, respectively.

**Table 2:** Prediction accuracy (non-parametric bootstrap 95% CI).

	10-min interval	Specificity (%), true ICP = ”1”		Sensitivity (%), true ICP = ”2”	
		General^a^	Enhanced^b^	General^a^	Enhanced^b^
Probability of predicting the true ICP level (%)	First	96.0 (95.8; 96.2)	95.0 (94.9; 95.1)	85.4 (83.6; 85.1)	87.1 (86.3; 87.7)
	Second	96.1 (95.9; 96.3)	94.7 (94.5; 94.9)	74.7 (73.5; 75.8)	80.6 (80.1; 81.2)
	Third	95.6 (95.5; 95.7)	93.8 (93.6; 93.9)	72.1 (69.8; 75.6)	75.7 (74.8; 76.7)
	Fourth	95.5 (95.3; 95.7)	93.0 (92.9; 93.2)	67.4 (66.3; 68.4)	75.0 (74.3; 75.9)
	Fifth	95.2 (95.0; 95.5)	93.3 (93.0; 93.4)	67.3 (66.0; 68.6)	73.0 (72.4; 73.6)
	Sixth	95.1 (94.9; 95.2)	93.9 (93.6; 94.1)	67.0 (66.3; 67.8)	72.7 (72.1; 73.4)

^a^General predictions based on maximum likelihood estimation. ^b^Enhanced predictions obtained with$\alpha =0.60.$

**Table 3:** Enhanced weighted mean prediction accuracy for individual patients (range of variation), $\alpha =0.60$.

	10-min interval	First	Second	Third	Fourth	Fifth	Sixth
Prediction accuracy (%)	True ICP = ”1”	95.3 (55; 100)	93.0 (33; 100)	92.2 (33; 100)	91.9 (25; 100)	90.4 (25; 100)	90.1 (25; 100)
	True ICP = ”2”	88.7 (21; 97)	83.2 (10; 95)	79.6 (10; 97)	78.4 (10; 97)	75.4 (10; 96)	73.0 (10; 94)

## Discussion

This paper describes and evaluates a simple and explainable probabilistic model for prediction of ICP levels of individual patients with severe TBI during treatment in the ICU. Predictions were made for the next hour to come, based only on the past hour of ICP variation of that patient. A method for prediction enhancement was also presented and evaluated, showing that a simplistic method for prediction probability weighting could compensate for imbalanced data and considerably improve prediction accuracy.

### Prediction accuracy and clinical applicability

For the prediction task ICP ≥ 20 mmHg, the model presented in this study had a very high specificity (93.9–95.0) and a good to high sensitivity (72.7–87.1). Results were similar (specificity 90.1–95.3 and sensitivity 73.0–88.7) when the model was trained based on the leave-one-out cross-validation method and evaluated on individual patients, which is important since this will be the case in the clinical situation. The wide ranges of variation when predictions were made for individual patients uninterruptedly are a result of the very few instances of severe ICP in some subjects. Low prediction accuracy for rare occasions is a very common aspect of most prediction models. For both models the prediction accuracy decrease the further away from the current time point the prediction was made. This is reasonable since the recent past is likely to be more informative of the future, but this shows the need to determine a threshold between the time needed to take necessary actions in the ICU to avoid secondary insults, as opposed to having too low prediction accuracy for time periods further ahead.

Gűiza et al.^9^ were the first to build a model to predict future ICP. They used multiple logistic regression and Gaussian processes to predict continuous ICP > 30 mmHg for at least 10 min 30 min in advance based on 4 h of past ICP and ABP. Their model had a classification accuracy of 77%, a sensitivity of 82% and a specificity of 75% in the first validation cohort. The model was later validated on two other cohorts of adult TBI patients; 151 subjects part of a multicenter study including five centers^21^ and 257 other subjects recruited as part of the CENTER-TBI high-resolution ICU monitoring cohort^22^. The precision varies somewhat between the cohorts (sensitivity/specificity 70/90% and 83/91% respectively) but are overall in the same range as the ones found with our model.

Recently, an online machine learning and signal processing framework that forecasts onsets of acute intracranial hypertension up to 8 h in advance using waveforms of different physiological signals was introduced^23^. Their model had a sensitivity of 90% but the precision was only 30% based on data from a waveform database. The study showed the importance of information contained in high-frequency waveforms in neurological signals, that could motivate future studies on pre-hypertensive patterns and the design of new alarm algorithms for critical events in TBI patients.

There are also other model applications that did not reach as high prediction accuracy, i.e., a prediction of prolonged intracranial hypertension based on morphological waveform features computed from ICP variations^15^. Here, ICP > 20 mmHg was predicted for time-to-onset between zero and 10 min with a sensitivity/specificity of 52/72% for predictions made six minutes ahead.

From a clinical perspective it has been shown that ICP levels exceeding 20 mmHg will result in worse outcome. This observation was already reported in 1977 in patients with TBI^24^ and many studies have confirmed it since, recently in the multicenter CENTER-TBI setting^25^. All treatment guidelines for TBI also state that ICP should be kept under about 20 mmHg. Actions that may be taken to prevent a dangerous ICP elevation include optimization of homeostatic and respiratory parameters, positional change of the patient, CT investigation to rule out space occupying lesions suitable for surgical intervention and treatment of acute hydrocephalus in need of external ventricular drain. These measures are applicable within the heads-up warning time presented in our study.

### Probabilistic model

The conditional probabilities (parameters) of the current model are estimated with the method of maximum likelihood, i.e., they have closed form solutions and no assumptions of statistical models are needed. These kinds of estimates enable general predictions of future events, as opposed to standard Bayesian methods such as maximum a posteriori estimation where subjective opinions of the probabilities of different events are also needed, in addition to data. Discriminative parameter learning methods, such as maximization of conditional likelihood^26^ and minimization of prediction error measures (defined by weighting predictions appropriately)^19^ are also applicable to obtain higher prediction accuracies. However, such methods enhance prediction accuracies of all possible outcome levels, often resulting in only slight increases in overall prediction accuracies at the expense of computationally intense algorithms. Here, another advantage with the maximum likelihood method is that it enables enhancement of the more important category of the response variable alone and may be combined with our suggested prediction enhancement method without unnecessary complexity or computational burden.

As a comparison to our new probabilistic model we applied six different autoregressive (AR) models, one for each prediction interval, to the same data set. The prediction accuracies of those models ranged from 84.4 to 98.0% (specificity, true ICP = ”1″) and from 65.2 to 81.0% (sensitivity, true ICP = ”2″). Thus, these models also perform well to a certain extent, but in all time intervals they are somewhat inferior to our probabilistic model when it comes to predicting dangerously high intracranial pressure (ICP = ”2″). The AR models also have other weaknesses. Firstly, they cannot be expanded easily to include other time series data such as ABP, PRx, etc. Secondly, for these regression type models our enhancement method cannot be applied since they do not generate prediction probabilities but only do mean predictions with Gaussian assumptions. Thirdly, AR models cannot do predictions if some observations on the explanatory variables are missing whereas our models can, since they are not statistical models but probabilistic ones.

When developing a prediction model, there is always a trade-off between sensitivity and specificity. The level of the trade-off must be determined by the application at hand, and what kind of error is “tolerable” in the specific clinical situation. When monitoring ICP in patients with TBI, time frames with severely high ICP levels are much less frequent than those with normal levels, e.g., due to the treatment regimen applied to avoid high ICP levels. This results in highly imbalanced data with large amounts of “normal” and small amounts of the “abnormal” levels that are set out to be predicted. When such data are used for computational prediction model building, the prediction accuracies for minority categories are often dominated by those of majority categories, resulting in a clinically less valuable model. One strategy to avoid this problem in model building is either to downsample the majority categories or upsample the minority categories when forming the training data. However, this may introduce sampling bias into the training data, thus affecting the model explainability and accuracy. In this paper, a different strategy to handle the imbalanced data was applied. This approach is directed towards increasing the prediction accuracies of the minority categories which are clinically more important. Based on clinical experience, the correct prediction of ICP ≥ 20 mmHg was considered twice as important (w = 2) compared to erroneously predicting ICP ≥ 20 mmHg when it was truly < 20 mmHg, i.e., it was considered twice as important to be alerted of a possible pressure increase even though this might lead to some additional false alarms.

The selection of $\alpha$ is crucial. It can be selected subjectively or objectively depending on the application at hand. A method based on semi-objective criteria for total prediction accuracy gain $T$ was implemented in this study. Note that generally, ${f}_{1}\left(\alpha \right)\le 0$ and ${f}_{2}\left(\alpha \right)\ge 0$, e.g., one can define ${f}_{i}\left(0.60\right)$ as the difference between the mean prediction accuracy for ICP = i when $\alpha =1$ and $\alpha =0.60,$ for $i=1, 2.$ A mathematical way to select a value of $\alpha$ is to maximize the $T.$ In this study, $\alpha =0.60$ leads to considerable enhancement of the prediction accuracy for ICP = 2 at the expense of very little loss of prediction accuracy for ICP = 1. Predicting ICP = 2 when the true value is ICP = 1 may also often be towards the end of a spell of true ICP value of “2”. So, in practice such an error may be handled rather easily.

### General probabilistic model

The general model was introduced to show that it is easy to combine many variables, e.g., not only several time series but also static observations such as sex, age, medical scores, etc. into the prediction process to evaluate the predictive strength of different combinations of variables. The possibility to include many predictors is one of the main strengths of the proposed model, apart from its ease in implementation. Furthermore, this model can be used in various ways, e.g., based on different time scales, different statistics of the observations, etc. In this sense, it is a more flexible model than e.g., standard autoregressive time series models or even traditional neural network models, where raw observations are generally used as predictors. Time and frequency domain features and use of waveforms can reveal complex dependencies which may be valuable for the prediction task^27^. Use of such features in our general model is straightforward. However extensive modelling needs to be conducted to identify “best” feature variables. Ideally, such experiments should include large data sets from several centers to enable context independent models.

## Conclusion

A new model for predicting ICP during ICU treatment, based on past ICP only, was developed and evaluated. A new and easily implementable prediction enhancement method, to compensate for imbalanced data, was also presented. The prediction model is promising for further development into a tool offering a proactive warning system to avoid secondary insults and associated poor outcomes with regards to increases in ICP. The model can also be fully generalized for incorporation of other time series data or clinical parameters, thus making future studies of various combination effects possible.

## Data availability

The datasets used and/or analyzed during the current study are available from the corresponding author on reasonable request.

## Abbreviations

3Q: Third quartile
ABP: Arterial blood pressure
CI: Confidence interval
CPP: Cerebral perfusion pressure
ICP: Intracranial pressure
ICU: Intensive care unit
TBI: Traumatic brain injury
